# Stress in Caregivers and Children with a Developmental Disorder Who Receive Rehabilitation

**DOI:** 10.3390/children7090136

**Published:** 2020-09-15

**Authors:** Sung Hyun Kim, In Young Sung, Eun Jae Ko, Jieun Park, Nayoung Heo

**Affiliations:** 1 Department of Rehabilitation Medicine, University of Ulsan College of Medicine, Ulsan 44033, Korea; 0735159@uuh.ulsan.kr; 2 Department of Rehabilitation Medicine, Asan Medical Center, University of Ulsan College of Medicine, Seoul 05505, Korea; gonjae0610@gmail.com (E.J.K.); pje3117@naver.com (J.P.); nayoung.heo@amc.seoul.kr (N.H.)

**Keywords:** development disorder, stress level of children and caregivers, home rehabilitation, early rehabilitation

## Abstract

This study aimed to evaluate the stress levels of caregivers and children with developmental disorders who were receiving rehabilitation treatment. The relationships between stress levels and factors such as early rehabilitation and home rehabilitation were quantified. Methods: This study was conducted in children with development disorders, aged from 1.5 years to 18 years, who were undergoing rehabilitation. The Korean version of the Child Behavior Checklist (K-CBCL) and the Adult Self-Report (K-ASR) were used to evaluate stress levels in children and caregivers, respectively. Results: Questionnaires were provided to 150 caregivers who agreed to participate. However, only 76 copies of the K-CBCL and 75 copies of the K-ASR were collected. The mean K-CBCL and K-ASR *t* scores were in the normal range. The K-CBCL score correlated positively with the K-ASR score (*p* value < 0.5). K-CBCL externalizing problems score correlated positively with the age at the start of rehabilitation, and the K-CBCL and K-ASR externalizing problems scores correlated negatively with home treatment delivered by caregivers. Conclusions: Stress levels of children and caregivers were closely related. Home rehabilitation provided by caregivers reduced stress in both caregivers and children. Early rehabilitation did not impart additional psychological burden on caregivers or children.

## 1. Introduction

The life expectancy of pediatric patients has improved significantly with advances in pediatric medical care [1,2]. As a result, the number of pediatric inpatients with developmental disabilities who receive rehabilitation has increased [3]. Children in need of rehabilitation have various diagnoses, including cerebral palsy, developmental delay, genetic disorders, and traumatic brain injury. They typically require long-term support from their families, doctor, and physical therapist and have problems in motor development and cognitive, language, and social areas [4]. Therefore, the comprehensive rehabilitation needs of pediatric patients are increasing and the benefits of early intervention have been emphasized [5].

Children receiving rehabilitation can be stressed by many factors. Recently, early rehabilitation has been emphasized, and this could impart a psychological and physical burden [5,6]. The socioeconomic status of the caregiver and the frequency of rehabilitation treatment could affect the stress level of the child [7].

Previous studies have evaluated the psychological status and quality of life of caregivers of children with disability [8,9,10,11]. Rentinck et al. found that parents caring for a child with cerebral palsy had a higher level of stress and worse mental health than parents of children without a disability [12]. Caregivers of children with disabilities face many stressors and demands. Home treatment aims to involve the parents in providing rehabilitation for their child and varies according to the child’s needs. Doctors have emphasized the importance of home treatment by caregivers, especially for patients with cerebral palsy, because inpatient rehabilitation treatments are not feasible or affordable. Novak et al. reported that an occupational therapy home program delivered by parents was clinically effective in children with cerebral palsy [13].

Early intervention, performed during the first year of life when the brain is undergoing rapid development, is thought to be more effective than intervention delivered later in development. This is based on the perception that neural networks that remain intact after brain injury could be enhanced through learning-induced plasticity. Animal studies demonstrate an important period of motor plasticity and activity-dependent reorganization of the motor projection pattern before 1 year of age [14]. However, few studies have investigated the effects of early rehabilitation on psychological status in children.

In this study, our first objective was to investigate the stress levels of caregivers and children receiving rehabilitation. Our second objective was to explore the relationship between stress levels and both early treatment and home treatment.

## 2. Methods

### 2.1. Study Design and Participants

This was a prospective study conducted in children with development disorders who underwent rehabilitation at the Division of Pediatric Rehabilitation, Department of Rehabilitation Medicine, Asan Medical Center between June 2016 and September 2016. The children with development disorders were over 1.5 years of age and we obtained informed consent from their caregivers. The Korean version of the Child Behavior Checklist (K-CBCL), the Korean version of the Adult Self-Report (K-ASR), and the questionnaire were provided to caregivers. Age, diagnosis, and comorbid medical problems were obtained from medical records. The study was approved by the institutional review board of the hospital (IRB no. 2016-0049).

### 2.2. Measurements

The K-CBCL was used to evaluate the stress level of the child receiving rehabilitation. The K-CBCL is a Korean standardized form of the Child Behavior Checklist [15]; it is used widely to identify behavioral and emotional problems in children and in both research and clinical practice. It has been used to evaluate the stress levels of children [16,17,18,19] and was included in a large multicultural study due to its validation against the K-CBCL [20]. There are several versions of the K-CBCL. In this study, the K-CBCL 1.5–5 was used for children aged from 1.5 years to 5 years and the K-CBCL 6–18 was used for children aged from 6 years to 18 years.

The K-CBCL 1.5–5 consists of a 100-item checklist and the K-CBCL 6–18 consists of a 119-item checklist, both of which are scored on a three-point scale (0 = not true; 1 = somewhat or sometimes true; 2 = very true or often true). The K-CBCL 1.5–5 is grouped into seven subscales: emotionally reactive, anxious/depressed, withdrawn, somatic complaints, sleep problems, attention problems, and aggressive behavior. The emotionally reactive, anxious/depressed, somatic complaints, and withdrawn subscales are combined to give a score for internalizing problems (IP). The attention problems and aggressive behavior subscales are combined to give a score for externalizing problems (EP). The total problems (TP) score is obtained by combining all seven subscales (IP, EP, sleep problems) and other problems [21]. The K-CBCL 6–18 is grouped into eight subscales: anxious/depressed, withdrawn/depressed, somatic complaints, social problems, thought problems, attention problems, rule-breaking behavior, and aggressive behavior. The IP score is composed of three subscales (withdrawn/depressed, anxious/depressed, somatic complaints), the EP score is composed of two subscales (rule-breaking behavior, aggressive behavior), and the TP score is obtained by combining all eight subscales (IP, EP, social problems, thought problems, attention problems) and other problems.

The K-ASR was used to evaluate the stress level of the caregiver [21,22,23]. The ASR is a reliable and valid self-report tool suitable for individuals aged 18–59 years and was designed to assess the extent of a variety of emotional and behavioral problems in adults. The ASR is a 126-item checklist that uses a three-point scale. The items are grouped into eight subscales: anxious/depressed, withdrawn, somatic complaints, aggressive behavior, rule-breaking behavior, intrusive, thought problems, and attention problems. The IP score is composed of three subscales (anxious/depressed, withdrawn, somatic complaints), the EP score of three subscales (aggressive behavior, rule-breaking behavior, intrusive), and the TP score of all eight subscales (IP, EP, thought problems, and attention problems) [24].

For both the K-CBCL and the K-ASR, a higher raw score indicates more problem behaviors [22,25]. Raw scores were transformed into *t* scores that indicate whether or not the individual presents deviant behavior or deficiency competencies in relation to norms for their age and gender. The *t* scores for IP, EP, and TP were standardized based on the percentile scores obtained from theKorean population [26]. A *t* score ≤59 (84th percentile) was classified as normal, 60–63 (85–90th percentile) as borderline clinical, and ≥64 (91st percentile) as clinical [27].

The questionnaire was used to evaluate factors that may affect the stress levels of children and their caregivers. It evaluates the characteristics of the rehabilitation (number of sessions of per week, number of institutions involved, and total duration of any treatments delivered at home by the caregiver) and the socioeconomic conditions (education level of the caregiver, caregiver’s annual income, marital status, and whether or not they live with their child).

### 2.3. Statistical Analysis

Relationships between variables were evaluated using Spearman’s correlation coefficient. Each factor and stress index were assessed by Spearman’s correlation coefficient. A *t*-test was used to compare the mean scores of each stress index between children who received some treatment at home by their caregiver (home treatment group) and children who received no treatment at home by their caregiver (no home treatment group). A *p* value below 0.05 was considered statistically significant.

## 3. Results

### 3.1. Participant Characteristics

The demographic details of the children and caregivers are presented in Table 1. A total of 150 caregivers agreed to participate. The K-CBCL, K-ASR, and questionnaires were distributed personally. For the K-CBCL, K-ASR, and questionnaires, the response rate was 50.6% (76 completed questionnaires), 50.0% (75 completed questionnaires), and 48.6% (73 completed questionnaires), respectively. The low response rate may have been due to the time required to complete all questionnaires (approximately 1 h) or reluctance to answer all items within the questionnaires. The 76 completed K-CBCL questionnaires comprised 81 K-CBCL 1.5–5 (80.2%) and 15 K-CBCL 6-18 (19.7%).

All participating children lived with their parents. The mean ± standard deviation age of the children was 4.4 ± 3.1 years (range, 1.5–16 years). Thirty-three (43.4%) children had cerebral palsy, 18 (23.7%) had a genetic disorder, 16 (15.8%) had delayed development of unknown etiology (Table 1). The number of rehabilitation treatments per week was 6.2 ± 3.6 (range, 1–14), delivered at 2.8 ± 1.3 institutions (range, 1–6). Forty (53%) children received rehabilitation treatment from their caregiver at home. Twenty-six (34.2%) children had started rehabilitation before the age of 6 months, 22 (28.9%) had started between the ages of 6 and 12 months, 14 (18.4%) children had started between the ages of 12 and 24 months, and 14 (18.4%) had started after the age of 24 months (Table 1).

The mean age of the caregivers was 36.7 ± 3.62. One caregiver was divorced. Most of the caregivers were female (98.7%) and college graduates (71.2%) and had cared for their child for more than 3 years (46.6%).

### 3.2. Stress Levels of Children

The mean K-CBCL TP *t* score was 54.1. Median IP and EP *t* score was 52.8 and 52.5, respectively. Among the 76 children, seven (9%) were in the clinical range for the TP score and 12 (16%) were in the borderline clinical range. Four children (5%) were in the clinical range for the IP score and 10 (13%) were in the borderline clinical range. Seven children (9%) were in the clinical range for the EP score and nine (12%) were in the borderline clinical range (Table 2).

### 3.3. Stress Levels of Caregivers

The mean K-ASR TP *t* score was 49.0. Six caregivers (8%) were in the clinical range for the TP score and three caregivers (4%) were in the borderline clinical range. Two caregivers (3%) were in the clinical range for the IP score and 12 (16%) were in the borderline clinical range. Five caregivers (6%) were in the clinical range for the EP score and two (3%) were in the borderline clinical range (Table 2).

### 3.4. Relations between Measured Factors

**Figure 1:**
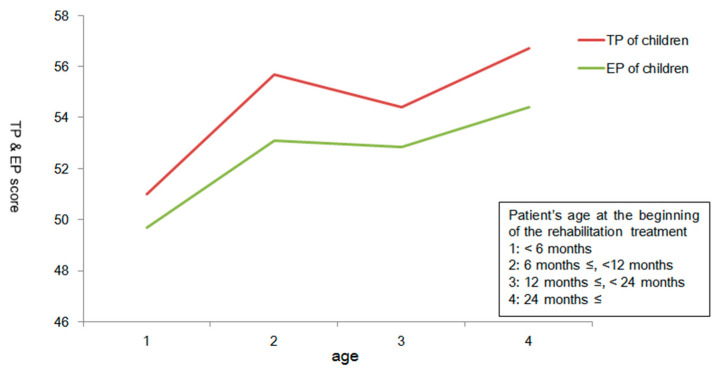
The relationship between the timing of rehabilitation and child’s stress level. EP: the externalizing problems are obtained by combining attention problems and aggressive behavior. TP: the total problems score is obtained by combining all eight subscales.

The K-CBCL TP score correlated positively with the K-ASR TP, IP, and EP scores, the duration of caregiving, and the age at which rehabilitation was started (Figure 1). Figure 1 displays the relationship between the timing of rehabilitation and child’s stress level of TP and EP. The K-CBCL TP score did not correlate with the number of rehabilitation sessions per week, the number of institutions across which rehabilitation was delivered, or socioeconomic status. The K-CBCL EP score correlated positively with the K-ASR TP, IP, and EP scores, the duration of caregiving, and the age at which rehabilitation was started (Figure 1). The K-CBCL IP score correlated positively with the K-ASR TP, IP, and EP scores but not with any other measured variables (Table 3). The K-CBCL EP score and the K-ASR EP score differed across the home treatment and no home treatment groups (Table 4). Table 4 shows that home treatment affects the EP scores of children and caregivers. In addition, children receiving home treatment had a higher number of rehabilitation sessions per week.

Externalizing problems score has a significant difference between the groups, and a *p* value below 0.05 was considered statistically significant.

## 4. Discussion

We evaluated stress levels in caregivers and children who received rehabilitation and evaluated factors that could affect stress levels. We found that the stress levels of children receiving rehabilitation correlated positively with the stress level of the caregiver. The stress levels of the children and their caregivers were lower if the child was receiving some rehabilitation treatment at home than if they were receiving no treatment at home but did not correlate with number of rehabilitation sessions per week or socioeconomic status.

Some of the children in this study reported remarkably high levels of stress. The proportion of children in the clinical range was 9%, 5%, and 9% for TP, IP, and EP, respectively, which is similar to the 10% expected in the general population [28]. In a previous study, the mean CBCL *t* score of non-fostered children was 51.7 [29], which is slightly lower than the mean *t* score in our study (54.1). However, when compared with the general population, our study group could be regarded as normal.

There have been previous studies of psychological problems in caregivers but fewer studies on the affected children [10,30,31,32,33]. There are some studies of the psychological problems of children with various diseases [34,35,36,37], particularly cerebral palsy [35,38,39,40,41]. Pain intensity, pain anxiety, parental stress and support, executive function, gross motor function, poorer intellect, and having disabled siblings are associated with psychological problems [35]. Parkes et al. evaluated psychological problems in children aged 8–12 years with cerebral palsy using the total difficulties score, which represents behavioral and emotional symptoms [35]. Of the parents who reported that their children had psychological problems, 95% said they also had family burden over a year.

Although studies have evaluated the psychological status of children receiving rehabilitation (but not their caregivers), few have done so simultaneously. Spiel at al. investigated parental stress and behavioral problems in children with cerebral palsy [38]. Moo’s life stressors and social resources inventory was used to evaluate caregiver stress and behavioral problems were identified using the CBCL. The CBCL IP score of children with cerebral palsy was associated with the situational stress of their caregivers, a measure which included health, economic situation, and life events [38]. The CBCL EP score of children with cerebral palsy was significantly associated with the situational stress and relational stress of their caregivers; the latter included social relationships with partners, family, friends, neighbors, and teachers [38].

In the present study, we found that home rehabilitation treatment was significantly associated with the EP score of children and their caregivers. This indicates that home rehabilitation treatment delivered by the caregiver may reduce stress in both the caregiver and the child. Based on the concept that parents are the principal component of the rehabilitation treatment team, our hospital has encouraged and educated all caregivers about home rehabilitation treatment. The therapist directly trained parents about the rehabilitation treatment appropriate for their children. Forty out of the 76 educated caregivers who completed questionnaires conducted home rehabilitation. Twenty-six of these performed home rehabilitation for 30 min every day, and 14 provided more than 30 min per day. The importance of home rehabilitation has been emphasized in various fields [42,43,44,45,46] and has been studied in children with cerebral palsy [13]. Novak et al. demonstrated that children with cerebral palsy who received occupational therapy at home had better occupational performance after 8 weeks than those who did not receive occupational therapy at home [13]. The psychological status of children was evaluated using the Children’s Assessment of Participation and Enjoyment, and there was no significant difference in psychological status between children who did and did not receive therapy at home. Claudio et al. found that caregiver-directed home-based intensive bimanual training in children with unilateral spastic cerebral palsy improved dexterity and performance of a functional goal [47]. However, this study did not investigate psychological status.

Early rehabilitation is helpful for the development of children, but it is not clear how it affects psychology. It may be a stress burden for caregivers and children to start rehabilitation too early. The benefit of early rehabilitation interventions for preterm infants has been emphasized recently [48]. The expected outcome of a developmental care intervention is improvement in overall physical, social, cognitive, and emotional development. However, we have not be able to find out how early rehabilitation treatment could affect psychological status in children with development disorder. In the current study, we identified the age of the child at the onset of rehabilitation to determine whether early rehabilitation treatment was related to stress in children with development disorders. Children who started rehabilitation early in their life had low TP and EP scores. In other words, early rehabilitation did not impart a psychological burden on children or their caregivers.

This study has several strengths. Firstly, we determined the effect of home rehabilitation delivered by caregivers on indices of stress. Home rehabilitation provided by the caregivers was associated with lower stress levels for both the caregiver and the child. Secondly, we demonstrated that early rehabilitation intervention did not increase the stress levels of children or their caregiver. Thirdly, we evaluated the stress levels of children receiving rehabilitation and their caregivers simultaneously and demonstrated that the two were correlated. Finally, a previous study evaluated the psychological burden of children with cerebral palsy, but our subjects included a variety of diseases.

The study has some limitations. Firstly, the sample size was small. The length of time required to fill out the questionnaire and the inclusion of sensitive issues likely contributed to the low response rate. Secondly, there was no control group, i.e., a group that received no rehabilitation, with which to compare the stress of children with development disorders. However, as children with development disorders were all receiving rehabilitation, it was not possible or ethical to include this control group. Moreover, the one of the principal limitations is the heterogeneity of the subjects. Children’s disease and age might affect their stress, so this study might have some bias in this regard. Therefore, well designed future research with homogeneity of pathology and age are needed. Moreover, disease severity was not studied; it is necessary to reflect upon how the severity of the disease affects stress in children and their caregivers.

In conclusion, this study demonstrated that the stress levels of children with developmental disorders were closely related to the stress levels of their caregivers. Early rehabilitation intervention did not impart an additional psychological burden to the caregiver or the child. Home rehabilitation provided by the caregiver was associated with lower stress levels of EP for of both the caregiver and the child.

## Figures and Tables

**Table 1:** Characteristics of the study subjects.

Characteristics of Children	
Number of children	76
Age (years)	4.4 ± 3.1
Sex (F:M)	42:34
Diagnosis	
Cerebral palsy	33
Genetic disorder	18
Developmental delay, unknown etiology	16
Intellectual disability	6
Other disease	3
Number of rehabilitation sessions per week	6.2 ± 3.6
Number of institutions involved in the rehabilitation	2.8 ± 1.3
Home treatment provided by caregivers	40
Age at which rehabilitation was first initiated (n)	
<6 months	26
6–12 months	22
12–24 months	14
≥24 months	14
**Characteristics of Caregivers**	
Number of caregivers	75
Age (years)	36.7 ± 3.62
Sex (F:M)	74:1
Marital status	
Divorced	1
Married state	72
Socioeconomic status	
Education level	
High school graduate	14
College graduate	52
Graduate school graduate	7
Annual income	
<30 million won	11
30–50 million won	22
50–70 million won	20
≥70 million won	20
Period of caregiving	
<2 years	19
2–3 years	20
≥3 years	34

Values are presented as number or mean ± standard deviation. Abbreviations: F = female, M = male.

**Table 2:** Prevalence of behavior problems in children and caregivers.

		Normal	Borderline Clinical	Clinical	Total
Children	Total behavior problems	57 (75)	12 (16)	7 (9)	76
	Internalizing problems	62 (82)	10 (13)	4 (5)	76
	Externalizing problems	60 (79)	9 (12)	7 (9)	76
Caregivers	Total behavior problems	66 (88)	3 (4)	6 (8)	75
	Internalizing problems	61 (81)	12 (16)	2 (3)	75
	Externalizing problems	68 (91)	2 (3)	5 (6)	75

Values are presented as number (%).

**Table 3:** Correlations between all measured factors.

		TP of Child	IP of Child	EP of Child	TP of Caregiver	IP of Caregiver	EP of Caregiver	Number of Institutions Involved in Rehabilitation	Number of Rehabilitation Sessions Per Week	Annual Income of Caregiver	Education Level of Caregiver	Period of Caregiving	Age at the Start of Starting Rehabilitation
TP of child	Rho	-	0.786	0.877	0.360	0.389	0.322	0.221	0.208	0.125	–0.052	0.291	0.289
	*p*		0.000 *	0.000 *	0.002 *	0.001 *	0.005 *	0.060	0.078	0.293	0.660	0.013 *	0.011 *
IP of child	Rho	0.786	-	0.543	0.407	0.436	0.351	0.161	0.213	0.107	0.193	0.011	0.133
	*p*	0.000 *		0.000 *	0.000 *	0.000 *	0.002 *	0.175	0.070	0.366	0.101	0.929	0.251
EP of child	Rho	0.877	0.543	-	0.237	0.273	0.223	0.177	0.140	0.111	–0.113	0.386	0.244
	*p*	0.000 *	0.000 *		0.041 *	0.017 *	0.054	0.134	0.239	0.351	0.339	0.001 *	0.034 *
TP of caregiver	Rho	0.360	0.407	0.237	-	0.904	0.882	0.122	–0.011	–0.52	0.183	–0.75	0.177
	*p*	0.002 *	0.000 *	0.041 *		0.000 *	0.200	0.309	0.924	0.666	0.125	0.531	0.129
IP of caregiver	Rho	0.389	0.436	0.273	0.904	-	0.695	0.140	0.013	–0.035	0.187	–0.030	0.140
	*p*	0.001 *	0.000 *	0.017 *	0.000 *		0.000 *	0.239	0.917	0.773	0.116	0.802	0.233
EP of caregiver	Rho	0.322	0.351	0.223	0.882	0.695	-	0.008	–0.117	–0.116	0.156	–0.142	0.224
	*p*	0.005 *	0.002 *	0.054	0.200	0.000 *		0.945	0.329	0.331	0.190	0.233	0.053
Number of institutions involved in rehabilitation	Rho	0.221	0.161	0.177	0.122	0.140	0.008	-	0.621	–0.046	0.031	0.306	–0.040
	*p*	0.060	0.175	0.134	0.309	0.239	0.945		0.000 *	0.698	0.792	0.008 *	0.736
Number of rehabilitation sessions per week	Rho	0.208	0.213	0.140	–0.011	0.013	–0.117	0.621	-	–0.065	–0.059	0.145	–0.124
	*p*	0.078	0.070	0.239	0.924	0.917	0.329	0.000		0.586	0.617	0.220	0.297
Annual income of caregiver	Rho	0.125	0.107	0.111	–0.52	–0.035	–0.116	–0.046	–0.065	-	0.312	0.082	0.135
	*p*	0.293	0.366	0.351	0.666	0.773	0.331	0.698	0.586		0.007 *	0.488	0.254
Education level of caregiver	Rho	–0.052	0.193	–0.113	0.183	0.187	0.156	0.031	–0.059	0.312	-	–0.178	0.049
	*p*	0.660	0.101	0.339	0.125	0.116	0.190	0.792	0.617	0.007 *		0.132	0.682
Period of caregiving	Rho	0.291	0.011	0.386	–0.75	–0.030	–0.142	0.306	0.145	0.082	–0.178	-	–0.069
	*p*	0.013 *	0.929	0.001 *	0.531	0.802	0.233	0.008 *	0.220	0.488	0.132		0.561
Age at the start of rehabilitation	Rho	0.289	0.133	0.244	0.177	0.140	0.224	–0.040	–0.124	0.135	0.049	–0.069	-
	*p*	0.011 *	0.251	0.034 *	0.129	0.233	0.053	0.736	0.297	0.254	0.682	0.561	

* *p* value < 0.05 by spearman correlation analysis. IP: the internalizing problems are obtained by combining emotionally reactive, anxious/depressed, somatic complaints, and withdrawn subscales. EP: the externalizing problems are obtained by combining attention problems and aggressive behavior. TP: the total problems score is obtained by combining all eight subscales (internalizing problems (IP), externalizing problems (EP), social problems, thought problems, attention problems) and other problems.

**Table 4:** Stress levels in the home treatment group and the no home treatment group.

		Children Receiving Home Treatment (n = 43)	Children Not Receiving Home Treatment (n = 33)	*p*
Children	Total problems score	53.07 ± 8.47	55.36 ± 8.15	0.24
	Internalizing problems score	53.28 ± 6.92	54.24 ± 6.99	0.52
	Externalizing problems score	50.47 ± 6.63	54.94 ± 8.31	0.03 *
Caregivers	Total problems score	47.70 ± 11.05	50.84 ± 8.31	0.18
	Internalizing problems score	49.35 ± 9.58	52.94 ± 10.70	0.13
	Externalizing problems score	45.98 ± 10.93	51.25 ± 7.68	0.02 *
	Number of rehabilitation sessions per week	6.95 ± 3.88	4.97 ± 2.91	0.02 *

Values are expressed as the mean (± standard deviation) *t*-score. * *p* values < 0.05 compare the two groups.
